# Multiple myeloma: family history and mortality in second primary cancers

**DOI:** 10.1038/s41408-018-0108-1

**Published:** 2018-08-07

**Authors:** Subhayan Chattopadhyay, Hongyao Yu, Amit Sud, Jan Sundquist, Asta Försti, Akseli Hemminki, Kari Hemminki

**Affiliations:** 1 Division of Molecular Genetic Epidemiology, German Cancer Research Center (DKFZ), Im Neuenheimer Feld 580, D-69120 Heidelberg, Germany; 2 Faculty of Medicine, University of Heidelberg, Heidelberg, Germany; 3 Division of Genetics and Epidemiology, The Institute of Cancer Research, London, UK; 4 Center for Primary Health Care Research, Lund University, 20502 Malmö, Sweden; 5 Department of Family Medicine and Community Health, Department of Population Health Science and Policy, Icahn School of Medicine at Mount Sinai, New York, USA; 6 Center for Community-based Healthcare Research and Education (CoHRE), Department of Functional Pathology, School of Medicine, Shimane University, Shimane, Japan; 7 Cancer Gene Therapy Group, Faculty of Medicine, University of Helsinki, Helsinki, Finland; 8 Comprehensive Cancer Center, Helsinki University Hospital, Helsinki, Finland

Since cancer survival rates in general are increasing, second primary cancers (SPCs) account for an increasing proportion of the overall cancer burden. In some cancer registries they account for more than 20% of new diagnoses^1^. Contributing factors for SPCs may be multiple, including iatrogenic adverse effects of chemotherapy or radiation, increased surveillance and the same causes that influenced patients’ first cancers, including family history and environmental causes^2–4^. Chemotherapy and radiation induce DNA damage which increases the risk of SPCs, and therapy-associated immunosuppression could also play a role. Treatment for multiple myeloma (MM) involves intense chemotherapy and concerns about SPCs have been raised, particularly relating to the possible effects of lenalidomide and melphalan^5^. The impact of family history was recently shown in survivors of Hodgkin lymphoma with an excess of lung, colorectal, and breast cancers in survivors with a family history of these cancers^6^. The potential importance of family history is emphasized by the fact that about 50% of patients with first primary cancer have a first-degree relative diagnosed with some cancer^7^. This proportion was also high among patients diagnosed with MM, 61%^7^. The other cancers in family members were diverse; including chronic lymphocytic leukemia and colorectal and prostate cancers^8,9^.

In the present study we use the Swedish Family-Cancer Database, with two goals, first to assess the influence of family history on the risk of SPC, and second to estimate the influence of SPC on mortality in MM in family members^7^. A family history implies that the type of SPC (e.g., lung cancer) was the same cancer that was diagnosed in a parent or sibling (e.g. lung cancer).

## Methods

In the Swedish Family-Cancer Database the second generation ‘offspring’ was defined as individuals born after 1931 and their patents were defined as the parental generation. Another truncation of data was caused by the start of cancer registration in Sweden in 1958. The study included 25,787 MM diagnosed from 1958 to 2015; of these 5205 were diagnosed in the offspring generation with a median age at diagnosis of 62 years. Among MM patients 360 (6.9%) were diagnosed with SPC after a median follow-up time of 4 years. Among these 360, 246 (68.3%) had a first-degree family history of any cancer.

Relative risks (RRs) were assessed with incidence rate ratios, estimated with RRs regressed over a fixed effects generalized Poisson model. RRs for SPC were obtained by comparing incidence rates for SPC X in MM patients with rates for first cancer X in the background population of the database. Family history was defined among parents and siblings. Familial RRs were estimated by comparing incidence rates between MM patients diagnosed with cancer X as SPC and having a family history of cancer X against those diagnosed with first cancer X in the population; the reference rate was the same as above. Sex, age group, calendar-period, socio-economic status, and residential areas were treated as potential confounders and were adjusted for in the regression model. Follow-up commenced from diagnosis of MM and was terminated on SPC diagnosis, emigration, death, or end of follow-up period, i.e. 2015, whichever occurred first. Confidence intervals were calculated for 5%, 1% and 0.1% level of significance^10^. All cancer-related deaths were stratified into MM, SPC, and other causes, including cancers defined in death certificates and non-neoplastic causes of death. Additive and multiplicative interactions of family history and risk of SPC were tested as described^11^.

The study was approved by the Ethical Committee of Lund University. Analyses are performed in SAS v9.4; please contact the authors for codes.

## Results

Among 5205 MM patients, 360 (6.9%) were diagnosed with a SPC. Familial SPCs were compared to non-familial SPCs in Table 1, which lists all SPCs with at least two cases having the same (concordant) tumor in a parent or sibling. Ignoring the overlapping impact of more than one cancer in family, prostate cancer was the major contributor to the family history (20%) followed by colorectal (14%), breast (10%), bladder (5%), lung cancer, and skin SCC (4% both). In patients without a family history of cancer, the risk of SPC was increased for skin cancer (squamous cell carcinoma, SCC, RR = 2.58) and leukemia (RR = 4.55). For patients with a family history of cancer, even though case numbers were low, risks were significantly elevated in a trend test for colorectal (RR/familial = 2.10 vs. RR/non-familial = 1.01), prostate (RR/familial = 1.60 vs. RR/non-familial = 0.56), and skin SCC (RR/familial = SCC, 8.82 vs. RR/non-familial = 2.58). The trend test was of borderline significance (*P* = 0.061) for lung cancer (RR/familial = 5.40 vs. RR/non-familial = 1.13). The highest SPC risk was observed for MM patients with a family history of leukemia (RR = 9.14, only two cases). Patients with SPC with any familial cancer (*N* = 246) were 68.3% of all SPCs and the RR was 1.38 vs. 1.13, respectively (trend test *P* < 0.001). We tested interactions of significant family risks and risk of SPC and found a stronger than additive interaction for skin cancer (*P* = 0.04).

**Table 1:** Relative risks of SPCs among all multiple myeloma patients stratified over family

Cancer	At least 1 FDR with cancer			No FDR with cancer			Total			Trend test *P* value
	*N*	RR	95% CI	*N*	RR	95% CI	*N*	RR	95% CI	
Colorectum	7	**2.10**	1.00–4.41	27	1.01	0.69–1.47	34	1.13	0.81–1.58	0.033
Lung	3	***5.40***	1.74–16.75	10	1.13	0.61–2.10	13	1.38	0.80–2.38	0.061
Breast	4	1.13	0.42–3.01	24	0.93	0.62–1.39	28	0.95	0.66–1.38	0.176
Prostate	20	**1.60**	1.03–2.48	38	***0.56***	0.41–0.77	58	**0.72**	0.56–0.93	0.006
Melanoma	2	**5.04**	1.26–20.14	18	1.46	0.92–2.32	20	**1.57**	1.01–2.44	0.087
Skin (SCC)	4	***8.82***	3.31–23.52	31	***2.58***	1.81–3.67	35	***2.81***	2.01–3.91	0.029
Leukemia	2	***9.14***	2.29–36.55	32	***4.41***	3.11–6.24	34	***4.55***	3.25–6.37	0.093
All	246	***1.38***	1.22–1.57	114	1.13	0.94–1.36	360	***1.29***	1.17–1.43	<0.001

Bold, italics and underline indicate 5%, 1% and 0.1% level of significance

*FDR* first degree relative, *N* frequency, *RR* relative risk, *CI* confidence interval, *SCC* squamous cell carcinoma

In order to check for possible skewed patient recruitment based on the multiple applied conditions were plotted the patient accrual over the study period (Supplementary Figure 1). The diagram shows MM patients with SPC and with or without family history (246 and 114 patients) plotted by 5-year intervals of MM diagnosis. No skewing of case accrual was observed.

The total number of deaths by the end of 2015 was 2872 (55.2%) among 5205 patients; and the total number of deaths among 360 patients with SPC was 228 (60.6%). The proportion was equally high among 246 patients with familial SPC, of whom 146 (59.3%) had died. Kolmogorov–Smirnov test on proportion difference found no evidence of statistical difference (*P* > 0.05).

MM was the most common cause of death in patients without a SPC (83.4%, 2194/2629), with 16.6% of deaths due to other causes (data not shown). For MM patients with a SPC, the distribution of causes of death is shown in Table 2. MM was the leading cause with 38.7% of deaths, followed by SPC 35.8% and other causes (25.5%); among other causes the majority of deaths (62.9%) were due to non-neoplastic causes. The mortality of SPC varied between second cancer types. For second pancreatic cancer, all seven patients died of this cancer; more than half of MM patients died of SPC when it was lung or nervous system cancer or leukemia. Other causes were important for CUP as SPC which is due to the practice of rarely describing CUP as a cause of death^12^. Among 82 deaths in patients with SPC without a cancer family history, majority was due to MM (36.6%), closely followed by SPCs (34.2%). Kolmogorov–Smirnov test found no significant difference in proportion contribution by the different causes of death in patients with or without family history (*P* > 0.05).

**Table 2:** Causes of death distribution of multiple myeloma patients diagnosed with SPC

Cancer	MM		SPC^a^		Other causes	
	*N*	%	*N*	%	*N*	%
UAT	2	50.0	2	50.0	–	–
Stomach	–	–	4	100.0	–	–
Colorectum	8	33.3	11	45.8	5	20.9
Anus	–	–	1	100.0	–	–
Liver	2	33.3	3	50.0	1	16.7
Pancreas	–	–	7	100.0	–	–
Lung	3	13.6	15	68.2	4	18.2
Breast	6	42.9	1	7.1	7	50
Cervix	–	–	1	100.0	–	–
Ovary	1	50.0	1	50.0	–	–
Prostate	11	42.3	5	19.2	10	38.4
Kidney	3	37.5	3	37.5	2	25
Urinary bladder	5	41.7	3	25.0	4	33.3
Melanoma	7	58.3	3	25.0	2	16.7
Skin (SCC)	16	72.7	1	4.5	5	22.7
Nervous system	3	42.9	4	57.1	–	–
NHL	5	45.5	4	36.4	2	18.2
Hodgkin lymphoma	–	–	1	50.0	1	50
Leukemia	7	24.1	16	55.2	6	20.6
CUP	3	21.4	1	7.1	10	71.4
Total^b^	94	38.7	87	35.8	62	25.5

*MM* multiple myeloma, *SPC* second primary cancer, *UAT* upper aerodigestive tract, *SCC* squamous cell carcinoma, *NHL* non-Hodgkin lymphoma, *CUP* cancer of unknown primary

^a^Cases noted only when at least one death is observed due to second cancer

^b^Total includes all cancers without constraints

## Discussion

The novel aspect of this study was the demonstration of the impact of familial risk on SPCs in MM patients. Accordingly, as many as 68.3% of SPCs were familial, i.e., a parent or sibling of MM patients were diagnosed with any cancer, moderately higher compared to that of 59.9% patients without an SPC. For three SPCs with significant risks, including colorectal, prostate, and skin cancers, the family members had exactly the same cancer as was the SPC. It is interesting that in a recent study from this database the most consistent familial association between MM and first primary cancers included colorectal and prostate cancer and leukemia^9^. This may not be coincidental and shared susceptibility may contribute to these findings. We showed also that MM patients with SPC appeared to have moderately worse prognosis (60.6% dead) compared to all MM patients (55.2% dead), while family history of SPCs did not increase mortality (59.3% dead). The limitation of the study was a relatively small sample size in spite of nation-wide coverage. The reason is that survival in MM, although improving, is still relatively poor whereby the time-window for SPCs is narrow^13^. Due to the small numbers we did not undertake formal hazard ratio analysis for survival.

Therapy-related SPCs in MM have mainly been associated with acute myeloid leukemia, which has been increased also in a recent study on German and Swedish MM patients^5,14^. The Swedish population of that study partially overlaps with the present one, where a risk (RR 4.41) of second leukemia was observed in patients lacking family history. Therapy-related side effects are still considered relatively weak in MM but the situation may change when larger patient groups achieve long survival times^5^. Family history needs to be considered a possible confounder in therapy-related studies on SPCs.

In conclusion, 68.3% of MM patients with SPC had a family history of any cancer. Significantly increased associations were found for second colorectal, prostate and skin cancers and family members diagnosed with these cancers. With continued therapeutic successes in MM treatment SPCs will be receiving increasing attention, whereby the contributing role of family history deserves inquiry into its mechanistic underpinnings.

## Electronic supplementary material

Supplementary Figure 1
