# Supplementary material for: Multiple myeloma: family history and mortality in second primary cancers
Source: Blood Cancer J. 2018 Aug 7;8(8):75. doi: 10.1038/s41408-018-0108-1 (PMC6081451; doi:10.1038/s41408-018-0108-1)
Supplement: Supplementary file 1 — Supplementary Figure 1 [file 41408_2018_108_MOESM1_ESM.pdf]

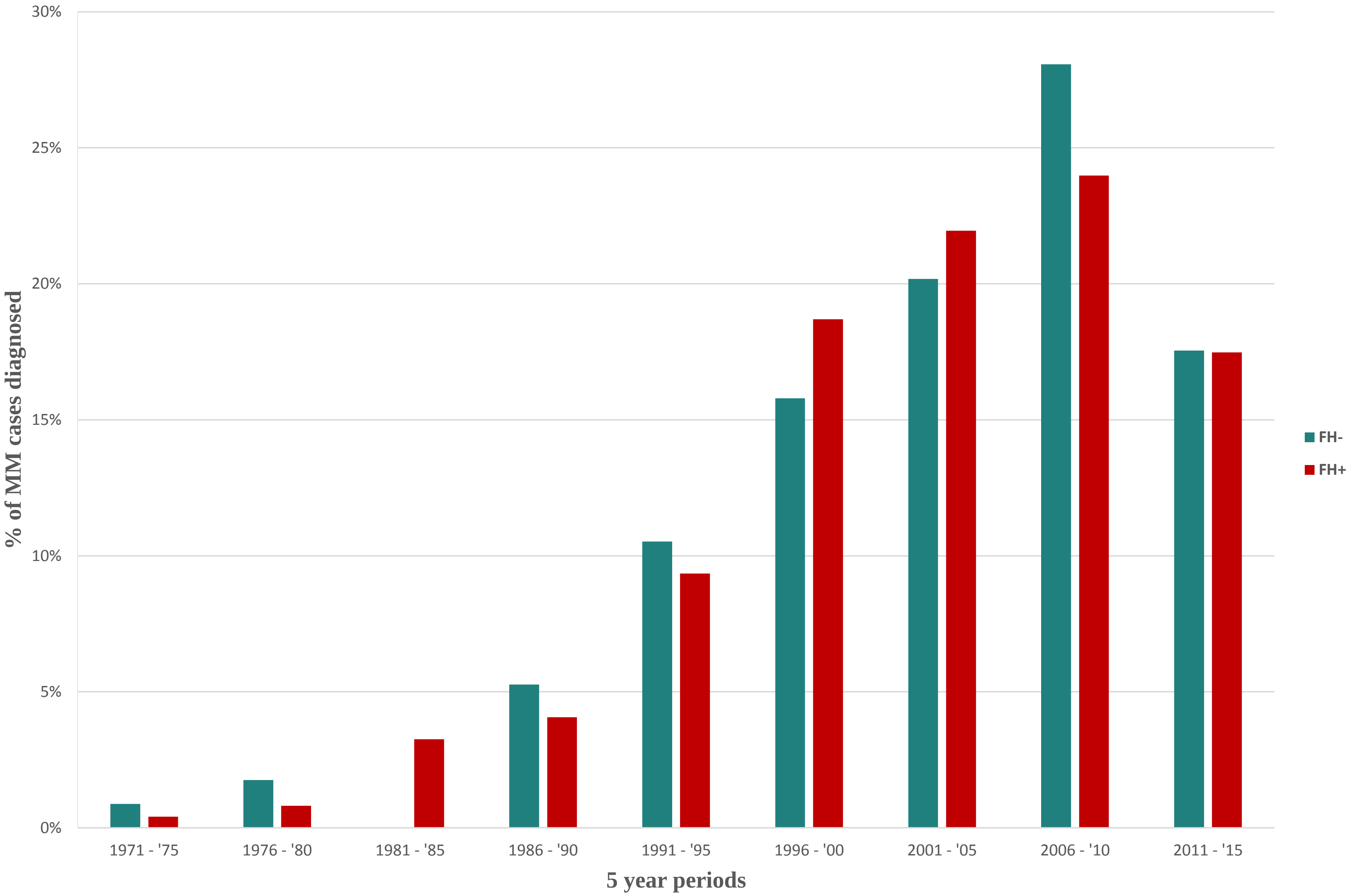


**Supplementary Figure 1** MM patients with SPC and with or without family history (246 and 114 patients) in 5-year intervals of MM diagnosis. FH-, family history negative and FH+, family history positive.
